# Developmental antecedents of young adults’ solidarity during the Covid‐19 pandemic: The role of sympathy, social trust, and peer exclusion from early to late adolescence

**DOI:** 10.1111/cdev.13660

**Published:** 2021-08-31

**Authors:** Jeanine Grütter, Marlis Buchmann

**Affiliations:** ^1^ Jacobs Center for Productive Youth Development University of Zurich Zurich Switzerland

## Abstract

This study explored characteristics of young adults’ solidarity during the Covid‐19 pandemic by identifying three different profiles, characterized by low (23%), average (54%), and high solidarity (23%). Based on longitudinal Swiss panel data (*N*_T1_ = 797, *M*_age T1_ = 12.15 years, 51% female; 28% migration background representing diverse ethnicities; *N*_T2_ = 707, *M*_age T2_ = 15.33 years; *N*_T3_ = 596, *M*_age T3_ = 18.31 years), the study combined person‐ and variable‐centered approaches to examine whether sympathy, social trust, and peer exclusion at earlier phases in development predicted membership in pandemic‐related solidarity profiles (*N*_T4_ = 300, *M*_age T4_ = 20.33 years). All developmental predictors were significantly associated with the likelihood of expressing solidarity during the pandemic as young adults.

**Abbreviations**
AIC: Akaike information criterion
BIC: Bayesian information criterion
FIML: full information maximum likelihood
GMM: growth mixture model
LMR: Lo–Mendell–Rubin
MAR: missing at random
SARS: severe acute respiratory syndrome
SEM: structural equation model

On March 11, 2020, the World Health Organization (2020) declared the novel coronavirus disease Covid‐19 a pandemic when it spread to more than 180 countries, prompting governments to introduce pervasive health measures and (partial) lockdowns (Brauner et al., 2020). The extent to which this global crisis is disrupting daily lives, introducing economic and social hardship as well as affecting psychological well‐being, varies by social groups (e.g., Ranta et al., 2020). Young adults may bear the brunt of the social distancing and confinement measures. Severe limitations of social contacts, including school closures (Lee, 2020), may be critical for this age group, as they impede the development of autonomy and compromise the sense of belonging, simultaneously placing high demands on their mental health (Andrews et al., 2020; Alonso‐Stuyck et al., 2018; La Greca & Harrison 2005; Van de Groep et al., 2020).

However, while having low risks of Covid‐19 health complications, young adults play a key role in the confinement of the spread of Covid‐19. They have been identified as a source of transmission, while often being asymptomatic. Hence, young adults may spread the virus without being aware of it (Davies et al., 2020; Ghosh et al., 2020), putting others, especially vulnerable ones, at risk. Therefore, it is important to learn whether there are different groups of young adults, willing to make sacrifices for the common good, and thus, expressing pandemic‐related solidarity.

The current study aims to shed light on how pandemic‐related solidarity is expressed among young adults, and which characteristics of this multidimensional construct reflect groups of young adults displaying high solidarity. From a policy perspective, it is not only important to learn whether pandemic‐related solidarity indeed reflects a multidimensional construct but also to illuminate the role of developmental antecedents that may later shape high solidarity during times of crisis. This study, therefore, investigates the role of social competencies developed across adolescence together with the role of peer relationships experienced during this developmental period for predicting solidarity profiles in young adulthood. In particular, sympathy and social trust developed from early to late adolescence (i.e., ages 12–18) and peer exclusion experienced during this time are examined for variation in solidarity responses. This approach provides insights into how and when specific developmental aspects can be fostered during different phases of adolescence in order to elicit desirable solidarity responses in times of future crisis. Today's young people will be tomorrow's adults, most likely confronted with other global crises (e.g., climate change), requiring well‐developed solidarity responses.

Research on young people's pandemic‐related solidarity is scarce. Most of the Covid‐19‐related work focused on their marked exposure to the social implications of the pandemic, particularly on the experience of emotional distress, mental health, and well‐being, shedding light on risk and protective factors (e.g., Ghosh et al., 2020; Shanahan et al., 2020). Some studies examined adolescents’ compliance with imposed mitigation measures, assessing factors that promote or encumber compliance (e.g., Alessandri et al., 2020; Nivette et al., 2021). Undoubtedly, compliance captures a critical component of pandemic‐related solidarity, which implies sacrificing individual freedom for the greater good of the community.

We argue, however, that solidarity in times of a pandemic goes beyond compliance with imposed measures. As a complex, multifaceted response, it is composed of *interrelated* risk perceptions, evaluation of health guidelines, compliance, as well as socio‐emotional, moral, civic, and peer‐related components. A rare study, capturing some solidarity components, examined longitudinally Dutch adolescents’ pandemic‐related values and altruism before and during the pandemic, and found stable levels of social value orientation and altruism measured during the imposed lockdown (Van de Groep et al., 2020).

The current study extends this prior work by examining pandemic‐related solidarity *profiles* in young adulthood (i.e., age of 20) and investigating how developmental precursors predict these profiles. Understanding the pandemic as a public goods dilemma (Van Lange et al., 2013), the study drew from the relational developmental systems’ metatheory (Lerner et al., 2014). In particular, it was proposed that solidarity in a pandemic may be framed, from a developmental perspective, as a mutually beneficial individual–context relation, embodying *adaptive* developmental regulation in young adults.

## Solidarity during a pandemic as adaptive developmental regulation

To understand individual responses to this global health crisis, we framed the pandemic as a public goods dilemma, when a shorter‐term loss may lead to a longer‐term gain (Van Lange et al., 2013). In this case, the longer‐term gains are the curbing of the virus and the alleviation of the economic, social, and psychological consequences of the crisis. The shorter‐term losses include constraints on individual freedoms and social connections. Individual preparedness to bear such restrictions for the sake of increasing the greater common (public) good (Yamagishi & Cook, 1993) reflects solidarity, a feeling of holding together, and an intention to stand in for each other (Bierhoff & Küpper, 1999).

Drawing from the relational developmental systems’ metatheory (Lerner et al., 2014), pandemic‐related solidarity can, thus, be conceptualized as individual–context exchanges assumed to be mutually beneficial. From this perspective, such exchange relations are beneficial to society, as solidarity reflects concerns for the welfare of others and the functioning of institutions. Likewise, they are beneficial to the solidarity‐expressing individual, as they lower risks of infection. Hence, expressing (high) solidarity in times of crisis mirrors *adaptive* developmental regulation, whereas the absence of (or low) solidarity indicates *maladaptive* developmental regulation.

The current study aimed to investigate how pandemic‐related solidarity is characterized among young adults and whether and how different components of solidarity would be interrelated for certain groups of young adults, assuming that these components would form distinct solidarity *profiles*. Conceptualizing solidarity as a multifaceted construct comprised of *interrelated* components, we posit that solidarity in a pandemic is composed of disease perceptions related to control and responsibility, evaluation of health guidelines, and compliance with measures (i.e., *disease‐related components*); moral judgments of pandemic‐related behaviors, concerns towards the vulnerable, and civic duties in the pandemic (i.e., *community‐related components*); and peer components (i.e., *pandemic‐related peer behavior*).

### Disease‐related components: Risk perceptions, evaluation of health guidelines, and compliance

Responses to a global infectious disease involve *the perception of whether the virus is controllable* (Wong & Tang, 2005) and whether it is within *one's responsibility of becoming infected* (Ho et al., 2005). They also include *evaluations of the effectiveness of Covid‐19‐related public health measures* introduced by the authorities (Gaygısız et al., 2012). These measures may be broadly categorized in *hygiene measures* (e.g., frequent hand washing and using hand disinfectant) and *social distancing measures* (e.g., keeping a distance of 1.5–2 m between persons not belonging to the same household; reducing social contacts; and staying at home; Alessandri et al., 2020). Young peoples’ evaluation of these measures is closely associated with *compliance* (Nivette et al., 2021). Risk perceptions, evaluation of measures, and compliance are expressions of solidarity insofar as they protect one's own health and that of others, thereby showing a sense of responsibility. They also imply partially sacrificing one's own personal rights in order to contribute to collective safety, thus investing themselves in the greater common good (Alessandri et al., 2020).

### Community‐related components: Concern, morality, and civic duties

Considerations for others and society are key to solidarity (Bierhoff & Küpper, 1999). In a pandemic, solidarity involves multifarious components of concern. Concern for those who are particularly exposed to risks and suffer from the pandemic amounts to caring for the needs of those who are struggling. This study, thus, included two concern‐related solidarity components, addressing *concern for others’ health* (Han et al., 2014) and *concern for people at risk of being infected*.

Times of crisis pose high calls on moral integrity and adherence to moral principles, as well as concerns about the welfare of others who are in need. Moral challenges during a pandemic also arise from testing an individuals’ motivation to act morally, adhere to moral norms, and distinguish what is right from what is wrong. Those who abide by moral rules have internalized norms of fairness (Turiel, 2006). These norms include *obligation judgments*, attesting moral wrongness to engage in unfair behaviors (Metzger et al., 2019), such as hording scarce resources in a pandemic. The moral wrongness lays bare as these actions serve one's own interest to the detriment of others, as it does in violating social distancing rules. The solidarity construct, thus, included two socio‐moral judgment measures: *hoarding resources* and *violating social distancing measures*.

As pandemic‐related solidarity revolves around contributing to the greater common good, it includes components reflecting civic competencies (Grütter & Buchmann, 2021) and volunteering (Neufeind et al., 2014). *Political efficacy beliefs*, encompassing external control beliefs about the responsiveness of the political system and its institutions (Beaumont, 2010), reflect, when applied to a pandemic, trust in the government in caring for the people and in responding appropriately to curb the spread of the disease (Gaygısız et al., 2012). Investing oneself in the common good during a pandemic also involves *volunteering* related to voluntary work for health services, grocery shopping for vulnerable people, or providing emotional support for them (Wolf et al., 2020; Wray‐Lake et al., 2017).

### Perceived pandemic‐related peer behavior and concern

As peer norms reflect consent of what is acceptable behavior in a given context (Veenstra et al., 2018), they may be of particular importance in shaping individual behavior during a pandemic, such as, compliance with social distancing rules (Andrews et al., 2020). Given the heightened need for social connection, young adults may be highly attentive to their peers’ behavior, using them as a reference for their own attitudes and behaviors (e.g., Blakemore, 2018). Accordingly, previous research documented positive peer influences on, for example, intentions to volunteer (Choukas‐Bradley et al., 2015) as well as negative ones with regard to health behavior and externalizing problems. This prior research assumed various mechanisms at work, such as conformity, peer pressure, and social sanctions on the one hand and social facilitation and learning on the other (for an overview, see Veenstra et al., 2018).

Against this background, the current study assumed that perceived peer behavior would inform adolescents’ perceived peer norms and composed a central component of young adults’ pandemic‐related solidarity, relevant for predicting group differences in their solidarity. Thereby, two aspects were considered in order to capture perceptions about peer behavior (i.e., *regarding social distancing*) and peers’ emotional reactions (i.e., *perceived peer concern with groups vulnerable to the virus*).

## Developmental antecedents of young adults’ solidarity during the Covid‐19 pandemic

Young adults’ pandemic solidarity involves mutually beneficial individual–context exchanges, reflecting adaptive developmental regulation. We assumed that *sympathy* and *social trust* developed from early to late adolescence are likely to facilitate mutually beneficial individual–context exchanges and, thus, promote adaptive developmental regulation as expressed in pandemic solidarity. Conversely, experiences of *peer exclusion* during this developmental period would make it more difficult to engage in such exchanges, thus, likely to provoke maladaptive developmental regulation as shown in low pandemic solidarity.

### Sympathy

Involving concerns for others’ welfare, sympathy is essential for motivating individuals to work toward meeting the needs of others (Eisenberg et al., 2014). The development of this other‐oriented competence may, thus, be a prerequisite for the capacity to engage in mutually beneficial individual–context relations that would later manifest themselves in pandemic solidarity. In this respect, research confirmed that sympathy is a developmental precursor for prosocial behavior in both childhood and adolescence (e.g., Malti et al., 2009), motivating actions that benefit others (Carlo & Padilla‐Walker, 2020).

Research also pointed to a normative developmental trajectory of sympathy with early formation, substantial growth in late childhood, and stabilization in early adolescence (Grütter & Buchmann, 2021; Zuffianò et al., 2018). This suggests that high levels of sympathy developed in early adolescence and remaining stable during this period may be conducive to adaptive developmental regulation during the pandemic, as expressed in high solidarity. We anticipated high solidarity for those with higher levels of sympathy and expected that sympathy in early adolescence would already be associated with later solidarity.

### Social trust

Social trust refers to beliefs that people can generally be trusted and are trustworthy (Alessandri et al., 2020; Uslaner, 2012). Research has not only shown that actions for the common good result in higher levels of social trust but also documented the inverse direction (Sønderskov, 2011). The underlying mechanism for the latter is that people are inclined to cooperate when they expect others to do so (Sønderskov, 2011, p. 66). These findings suggest that the development of social trust may be decisive for engaging in individual–context relations that are beneficial for all parties involved. People deeming others as trustworthy also tend to follow moral values and are less likely to engage in deviant behavior. This increases group solidarity and cohesion (Alessandri et al., 2020), likely to promote mutually beneficial individual–context relations. Hence, we assumed that high levels of social trust would be associated with adaptive developmental regulation as expressed in profiles higher in solidarity.

Developmental research has studied primarily interpersonal trust in familiar others, while longitudinal studies on developmental patterns of social trust in adolescence are still scarce (Wray‐Lake & Flanagan, 2012). Results converge, however, on the age‐related decline in social trust across adolescence, as young people acquire more differentiated views of others in light of the widening social world. At the same time, social trust also becomes more stable across adolescence, as representations of a generalized other crystallize (e.g., Flanagan & Stout, 2010). For this reasons, we explored whether trajectories characterized by higher trust and less decline would be associated with profiles higher in solidarity.

### Peer exclusion

Adolescents’ experiences of peer relationships are critical for their social development (Blakemore, 2018; Rubin et al., 2015): Experiences of social acceptance and supportive peer relationships foster prosocial and altruistic peer behavior (Stotsky et al., 2020), motivating adolescents to consider the well‐being of others. In contrast, peer exclusion can lead to social withdrawal longitudinally (e.g., reduced classroom participation; Buhs et al., 2006) and predicts internalizing and externalizing problems (Rubin et al., 2015). Regarding externalizing problems, rejected peers have been shown to be more likely to develop oppositional attitudes and deviant behavior and to be more susceptible for negative peer influences (Dishion & Tipsord, 2011). Based on these previous findings, we assumed that experiences of peer exclusion may make it more difficult to engage in social relations that are mutually beneficial, increasing the likelihood of maladaptive developmental regulation in later phases, deterring young adults from contributing to society, expressed in low pandemic‐related solidarity.

When considering developmental trajectories of peer exclusion across formal schooling (i.e., ages 5–18), research pointed to a decline in both prevalence and frequency (Ladd et al., 2017). Moreover, from ages 12 to 14, the share of adolescents belonging to the groups experiencing most negative peer relationships declined considerably (Nylund et al., 2007). We, therefore, explored whether trajectories displaying lower peer exclusion and decreases across time would be associated with higher solidarity.

## Current study

This study investigated Covid‐19‐related solidarity profiles in young adulthood (i.e., age of 20) and selected developmental antecedents from early to late adolescence (i.e., ages 12–18) in Switzerland. This country was among the first European nations hit by the Covid‐19 infectious disease, next to neighboring Italy (Shanahan et al., 2020). In March 2020, it belonged to the 10 most affected countries worldwide; its’ per capita rate of Covid‐19 infections being one of the highest (Salathé et al., 2020). On March 13, 2020, the Swiss government decided to close all schools and institutions of higher education, followed by a lockdown on March 16, 2020, whereby all non‐essential stores, restaurants, bars, and entertainment businesses were closed until April 27, 2020. Whenever possible, working from home was requested, social distances measures were enforced, gatherings of more than five people in public spaces were prohibited, and public transport was curtailed (Swiss Federal Office of Public Health, 2020). Mobility was cut back, as borders with neighboring countries were mostly closed. After April 27, 2020, measures were stepwise released, with primary schools reopening on May 11, 2020, followed by institutions of higher education on June 6, 2020. Data collection for this study took place within a 3‐week window starting in mid‐April 2020, 4 weeks into the lockdown. It ended on May 6, 2020, when the country was still in lockdown. Thus, the time window of collecting pandemic‐related data fell into the crucial period of the lockdown, hitting young people particularly hard in light of school closures and severe limitations on social gatherings.

As pandemic‐related solidarity involves a complex response, the study's aim was an exploratory one, identifying *solidarity profiles*. Using a person‐centered latent variable approach (i.e., latent profile analysis), we investigated whether and how the different components were interrelated for certain groups of young adults. We did not anticipate a particular number of profiles (Nylund‐Gibson et al., 2014), but we expected that profiles would be different in their level of expressed solidarity.

The second assumption was confirmatory, hypothesizing that sympathy and social trust developed from early to late adolescence (i.e., ages 12–18) and peer exclusion experienced in this period would represent developmental precursors for pandemic‐related solidarity profiles in young adulthood (i.e., age of 20). We argued that all components would motivate or discourage, respectively, engagement for the benefit of the common good, with young adults exhibiting higher levels of sympathy and social trust and lower levels of peer exclusion at earlier phases of development being more likely to belong to profiles of higher solidarity. For sympathy, we also expected that those with high levels at age 12 would be more likely to belong to higher solidarity profiles than those with lower levels, as sympathy has been shown to stabilize at this point (Grütter & Buchmann, 2021; Zuffianò et al., 2018).

Lastly, we anticipated that not all adolescents would develop in the same way, thus, investigating different growth trajectories of sympathy, trust, and peer exclusion (for a similar approach, see Nylund et al., 2007). Again, these analyses were exploratory, as we did not develop specific hypotheses about the number of growth trajectories. In particular, we explored whether adolescents characterized by higher initial levels (i.e., age 12) and either stable or increasing development (i.e., changes from ages 12 to 18) would be more likely categorized within profiles of higher solidarity at the age of 20.

In contrast to much Covid‐19‐related research, this study is not based on convenience sampling, but on a nationally representative cohort with multiple survey waves conducted prior to the health crisis.

## METHOD

### Participants and design

The data were collected within the COCON survey ‐ The Swiss Longitudinal Survey on Children and Youth Buchmann et al. (2021). The sample used for the current study consisted of the child cohort from the German‐speaking part of Switzerland, which is representative for this area. It was drawn in a two‐stage procedure, first selecting communities and then households from the community register. The study participants have been interviewed at various time points. For this specific study, the sample included 797 participants interviewed face to face in their homes at age 12 (51% female; 28% migration background; *M*_age T1_ = 12.15 years, *SD*_age T1_ = 0.23 years), 15 (*N* = 707, *M*_age T2_ = 15.33 years, *SD*_age T2_ = 0.19 years), and 18 (*N* = 596, *M*_age T3_ = 18.31 years, *SD*_age T3_ = 0.21 years). The latest data collection (*N* = 300) took place when participants were 20 years old (60% female; *M*_age T4_ = 20.33 years, *SD*_age T4_ = 0.19) via online survey (20 min). This survey spanned a 3‐week window in mid‐April of 2020, 4 weeks after the lockdown began in Switzerland. At the time of the survey, the majority of participants (61.7%) were enrolled in school and 23.8% were employed. Others were in an interim year (7.4%), in military/civilian service (4.3%), or unemployed (2.1%). When considering ethnicity and minority group status in the current study context, 27.7% of the children have a migration background among the sample of the 12‐years‐old (origins were former Yugoslavian states: 19.5%, Italy: 18.1%, Germany: 17.1%, Austria: 6.7%, other European countries: 22.4%, Asian countries: 9.5%, Northern American countries and the Caribbean: 2.4%, Latin American countries: 2.9%, and African countries: 1.4%). In addition, regarding parental education, in 31% of the sample at least one parent held a university degree.

This study was conducted in accordance with ethical standards of the APA and the Helsinki Declaration. In addition, the study's adherence to the Human Research Act (Swiss Federal Council, 2020) was monitored by the national funding agency. Before each interview at the ages of 12 and 15, caregivers provided their informed consent (i.e., written consent for the first survey wave, followed by detailed written information and oral consent before each subsequent survey wave). In addition, oral assent of the child was requested and they were able to withdraw from the study at any time. Before the adolescents participated in the study at the ages of 18 and 20, they were asked to provide their written consent. Parents and their children were informed that this study addressed the development of children in different life situations. For the last data collection, young adults were informed that the study investigated their perceptions and beliefs regarding Covid‐19 and how they responded to the lockdown measures and associated challenges. On the basis of 563 valid e‐mail addresses, participants were sent the information and link to the questionnaire (with two reminders being sent 1 week and 12 days after the survey started), of which 300 participants responded.

Regarding study attrition, the results showed that children from parents with higher parental education (odds ratio = 1.85, *p* < .001) and children without a migration background (odds ratio = 1.58, *p* = .032) were significantly more likely to remain in the study compared to children from parents with lower education and migration background. Therefore, missing at random (MAR; i.e., the missingness was related to observed variables) was supported (Enders, 2010) and missing data were accounted for with full maximum‐likelihood estimation (method: FIML) in *Mplus 8.6* (Muthén & Muthén, 2018).

### Measures

#### Components of Covid‐19 solidarity profiles

The solidarity profiles included 12 components, reflecting three dimensions: *disease‐related components*, *community‐related components*, and *perceived peer behavior*. All items and scales were adapted from previous research on these dimensions regarding the swine flu (e.g., Bults et al., 2011; Gaygısız et al., 2012; Rubin et al., 2009) or severe acute respiratory syndrome (SARS; Wong & Tang, 2005) and from previous research on moral judgments (Metzger et al., 2019) and civic engagement (Wray‐Lake et al., 2017). The three single‐item measures regarding disease‐related components have generated variance between participants in previous studies on risk perceptions and beliefs about the Swine flu or SARS and have been predictive of recommended protective behavior (e.g., Bults et al., 2011; Gaygısız et al., 2012; Rubin et al., 2009). All scales (i.e., mean scales) revealed high internal consistency in previous studies, as it did in the current study; descriptive statistics and reliability indices of the components are shown in Tables 1 and 2. For a full list of items for each construct, see Supporting Information (S1). Since Covid‐19 is mostly referred to as “Corona” in the Swiss media, this label was used in all items. If not specified differently, a 6‐point scale (0 = *completely disagree*, 5 = *completely agree*) was used.

**TABLE 1:** Descriptive statistics and correlations among the variables included in the latent profile at T4 (*N* = 300)

	*M* (*SD*)	(1)	(2)	(3)	(4)	(5)	(6)	(7)	(8)	(9)	(10)	(11)	(12)
1. Perceived control*	3.36 (1.12)	—											
2. Perceived responsibility	3.69 (0.95)	.21***	—										
3. Evaluation of measures	4.35 (0.56)	.29***	.32***	(.73)									
4. Non‐compliance with sd	1.78 (2.01)	−.09	−.08	−.19**	—								
5. Concern: others’ health	3.15 (1.02)	.08	.16**	.44***	−.16**	(.86)							
6. Concern for vg	4.02 (0.82)	−.02	.06	.26***	−.18**	.41***	(.85)						
7. MJ: social distancing*	3.52 (0.83)	.15**	.22***	.48***	−.44***	.37***	.21***	(.72)					
8. MJ: hoarding resources*	4.26 (0.80)	.15*	.05	.09	.09	.02	.02	.15**	(.46)				
9. Political efficacy beliefs	3.58 (0.75)	.28***	.18**	.38***	−.08	.16**	.20***	.30***	.12*	(.82)			
10. Volunteering	1.98 (0.89)	.05	−.02	.18**	−.13*	.23***	.22***	.19**	.01	.14*	(.61)		
11. Perceived peer behavior about sd	3.45 (0.93)	.03	.01	.15*	−.21***	.07	.14*	.16**	−.08	.25***	.10	—	
12. Perceived peer concern for vg	3.93 (0.73)	.06	.13*	.21***	−.14*	.27***	.64***	.23***	.07	.14*	.22***	.38***	(.94)

Note

Items, resp., scales with * have been recoded. Scale reliabilities (Cronbach's alpha) or correlations (for MJ behaviors with two items) are reported in the diagonals.

Abbreviations: MJ, moral judgment; Sd, social distancing; vg, vulnerable groups.

* *p* < .05.

** *p* < .01.

*** *p* < .001.

**TABLE 2:** Descriptive statistics and correlations among the predictor variables (*N* = 797)

	*M* (*SD*)	(1)	(2)	(3)	(4)	(5)	(6)	(7)	(8)	(9)
1. Sympathy T1	3.72 (0.70)	(.71)								
2. Sympathy T2	3.61 (0.62)	.38***	(.67)							
3. Sympathy T3	3.64 (0.62)	.36***	.45***	(.70)						
4. Social trust T1	2.82 (0.64)	.16***	.15***	.10*	(.51)					
5. Social trust T2	2.52 (0.63)	.08*	.15***	.16***	.37***	(.61)				
6. Social trust T3	2.49 (0.66)	.08	.15***	.18***	.29***	.51***	(.78)			
7. Peer exclusion T1	1.04 (1.02)	−.06	−.03	.06	−.27***	−.08*	−.08*	(.63)		
8. Peer exclusion T2	0.89 (0.76)	−.07	−.00	−.03	−.13***	−.12***	−.01	.35***	(.51)	
9. Peer exclusion T3	0.82 (0.61)	−.09*	−.07	−.10*	−.12**	−.05	−.07	.26***	.36***	(.49)

Note

T1 = age 12; T2 = age 15; T3 = age 18. Scale reliabilities (Cronbach's alpha) or correlations (for peer exclusion with two items) are reported in the diagonals.

* *p* < .05.

** *p* < .01.

*** *p* < .001.

#### Disease‐related components

##### Perceived control

This component was assessed with a single item: “Not much can be done against Corona,” adapted from an online questionnaire in the Netherlands about the swine flu (Bults et al., 2011). The negatively framed item was recoded.

##### Perceived responsibility

We adapted the item: “Whether I get Corona or not, depends on how I behave,” from a study on swine flu‐related beliefs (Gaygısız et al., 2012).

##### Evaluation of imposed measures

Participants evaluated how helpful certain strategies for preventing the spread of Corona were, which are as follows: “Meet fewer friends,” “Avoid crowds of people,” “Wash hands regularly and disinfect,” “Keep a distance of 2 m,” and “Stay at home” (0 = *very ineffective*, 5 = *highly effective*). The original scale, on which this measure was based, had been previously used in a study about beliefs regarding the swine flu pandemic (Gaygısız et al., 2012). For the current study, the four behaviors most relevant to the current study context were selected.

##### Non‐compliance with social distancing rules

This aspect was measured with the item: “How many friends have you personally met last week in total?” (during the lockdown, with higher numbers pointing to non‐compliance; adapted from Rubin et al., 2009).

#### Community‐related components

##### Concern for others’ health

Participants voiced their concern about others’ catching Covid‐19 by evaluating six items (e.g., “I am worried that I could infect my friends or my family with Corona” and “I am worried that, around the globe, many people will fall sick”), adapted from previous work about spreading SARS to the community (Wong & Tang, 2005).

##### Concern for vulnerable groups

The items to measure concern for vulnerable groups during the pandemic were adapted from Zhou et al. (2003). The scale consisted of five items (e.g., “I am concerned about elderly and sick people who severely suffer from the consequences of Corona”).

##### Moral judgment

Respondents rated whether it was wrong to perform certain activities related to Covid‐19. The original scale (Metzger et al., 2019) asked about adolescents’ civic involvement and whether it was wrong not to engage in certain activities. The content of the items was chosen based on two studies about the swine flu (Gaygısız et al., 2012; Rubin et al., 2009). *Moral judgments about violating social distancing measures* involved five items (e.g., “Is it ok or not ok if I: … take the bus or tram during my free time?” and “… to go to a party organized by my friends?”). *Moral judgments about hording scarce resources* were composed of two items asking whether it was ok or not ok if one bought more medicine or toilet paper, respectively, than needed (0 = *completely not ok*, 5 = *completely ok*). Both variables were recoded with higher values reflecting higher moral judgment.

##### Political efficacy beliefs during the pandemic

These beliefs were used in previous studies by Gaygısız et al. (2012) and Niemi et al. (1991). For the current study, the items were adapted to the Covid‐19 pandemic by using six items: (e.g., “The Federal Council has the situation under control” and “The Federal Council has issued rules that are too strict”), with negatively phrased items recoded.

##### Volunteering during the pandemic

Volunteering was assessed with the question “How often do you volunteer presently?” (adapted from Wray‐Lake et al., 2017) and included three items (e.g., “I help older people and other risk groups in the household” and “I help older people or risk groups by calling them to see how they are doing”; 0 = *I don't*, 4 = *daily*).

#### Perceived peer behavior and concern

##### Perceived peer behavior regarding social distancing during the pandemic

This aspect was measured with the item: “My friends follow the rules about social distancing released by the Swiss government,” adapted from a previous study about SARS (Wong & Tang, 2005).

##### Perceived peer concern for vulnerable groups

This construct was assessed with three items of one's own *concern for people at risk of being infected*, asking how respondents estimated their peers’ concern for others related to Covid‐19 (e.g., “My friends are concerned about elderly and sick people who severely suffer from the consequences of Corona”).

#### Adolescent predictors of the Covid‐19 profiles

The predictor scales (i.e., mean scales) were based on previously validated measures, whereby all items were rated on a 6‐point scale (0 = *completely disagree*, 5 = *completely agree*).

##### Sympathy

The scale (Zhou et al., 2003) consisted of four items: (e.g., “I feel sorry for children who cannot afford many things” and “I feel sorry for children who are being bullied”).

##### Social trust

Social trust was assessed with four items (e.g., “Most people take advantage of others when they have the opportunity” and “Most people think of their own advantage”; Deutsches Jugendinstitut und Infas Bonn, 2003). Negatively framed items were recoded.

##### Peer exclusion

Participants answered to the following two items: “I sometimes get picked on by peers” and “I sometimes get excluded by peers” (adapted from Buhs et al., 2006).

### Data analysis

The data analysis included a combination of person‐ and variable‐centered approaches, conducted in R and MPLUS. While variable‐centered approaches focus on explaining relations between variables, person‐centered approaches aim to explain relations among individuals (Jung & Wickrama, 2008).

First, for the person‐centered approach, we applied latent profile analysis in MPLUS with the goal to identify profiles (i.e., subpopulations within the samples) characterized by different patterns regarding a set of variables (Spurk et al., 2020). In our case, the aim was to find out how the different components used to measure solidarity combine into distinct solidarity profiles. We, thus, included all 12 indicators to identify the number of profiles. All of these indicators were entered in their original scales (i.e., continuous scales); however, since the items/scales were on different rating scales, all indicators were mean centered.

We compared solutions with different numbers of profiles (i.e., starting with 1 profile and increasing in +1 profile steps) and followed recommendations by Nylund‐Gibson et al. (2014) and Spurk et al. (2020). We used the Bayesian information criterion (BIC), whereby lower values indicate a better fit; the entropy value (i.e., the confidence with which individuals can be classified into a specific profile, ranging from 0 to 1, recommended >0.8); the Lo–Mendell–Rubin (LMR) test; and the bootstrap likelihood ratio test. Moreover, we looked at profile sizes and the interpretability of the different profiles, particularly how well the profiles differentiated between groups and whether they differed quantitatively (i.e., in the level) or also qualitatively (i.e., in the pattern). For all profiles, variances across profiles were freely estimated and covariances constrained to be unrelated to one another (i.e., constrained to 0 in order to facilitate model convergence). As a robustness check for the identified profiles, an additional sample of younger adolescents was investigated (i.e., to determine whether similar profiles could be identified).

Next, in order to address our hypotheses, whether sympathy, trust, and peer exclusion measured at earlier stages predicted profile membership at the age of 20, we chose two different statistical models, each controlling for different aspects. We ran the latent profile analysis with the predictors using the three‐step method with BCH weights as recommended by Asparouhov and Muthén (2014). This ensured that our latent profile variable was not affected by the predictor variables, and biased parameter estimates were avoided. Thereby, a multinomial logit model revealed the likelihood of belonging to one solidarity profile (chosen as the baseline category) over the other profiles, depending on each predictor (i.e., sympathy, trust, and peer exclusion entered as continuous variables at the ages of 12, 15, and 18). In this approach, however, we were not able to control for the stability of each predictor over time (i.e., from ages 12 to 18) and for the interrelatedness of the three predictors at each time point (i.e., the within‐time correlations).

Therefore, we ran a second model, whereby we combined our person‐centered approach with a variable‐centered one. We calculated a structural equation model (SEM), predicting latent profile membership by using numerical integration (i.e., Montecarlo integration in MPLUS). In both analyses, we were able to answer our developmental question at which prior ages sympathy, trust, and peer exclusion predicted profile membership in early adulthood. Importantly, the SEM was the most robust analysis, controlling for the relations of the predictor variables at each time point and for the rank‐order stability of each predictor variable over time.

Lastly, our final step was exploratory, aiming to identify growth mixture models (GMM), accounting for the possibility that there were specific subgroups in how individuals developed from early to late adolescence. In GMM, some parameters of traditional latent growth models are allowed to vary across subpopulations, whereby the subpopulations are identified based on different patterns of growth (Colder et al., 2001; Jung & Wickrama, 2008). Thus, instead of assuming a single trajectory, growth trajectories are assumed to be heterogeneous, whereby each latent growth trajectory class is defined by its unique growth model. To identify GMM for each predictor, we first predicted a traditional growth model by setting the intercept as a reference to the first assessment at age12 and specifying the slope (representing the change from ages 12 to 18) by 1 level units (i.e., 0, 1, 2, and 3). We also ensured that the condition of measurement invariance (i.e., scalar invariance) held, which is needed in order to reliably compare mean values (for details, see Supporting Information, S2). Then, we continued to specify a simple GMM with variances and covariances for the growth factors within each growth class fixed to zero (Jung & Wickrama, 2008). We started with a one‐class solution and increased the number of growth classes. The same criteria as for the identification of latent profiles applied. After identifying each GMM, we explored how the GMM profiles related to solidarity profile membership. Due to the relatively small sample size, these analyses were of descriptive nature and displayed in cross‐tables, whereby the overlap of the solidarity profiles with the profiles identified for each predictor was shown and differences investigated with *χ* ^2^‐tests.

When analyzing latent profiles and GMMs, the central assumption is that there are multiple subpopulations within a larger, more heterogeneous population; therefore, multiple imputation is not recommended as it assumes a single population (Colder et al., 2001). Thus, despite the small sample size, we controlled for missingness by using FIML in MPLUS. Since the missingness was related to observed variables, MAR was supported, which controls for biased estimations under the MAR assumption (Enders, 2010).

## RESULTS

### Latent profile analysis

**FIGURE 1:**
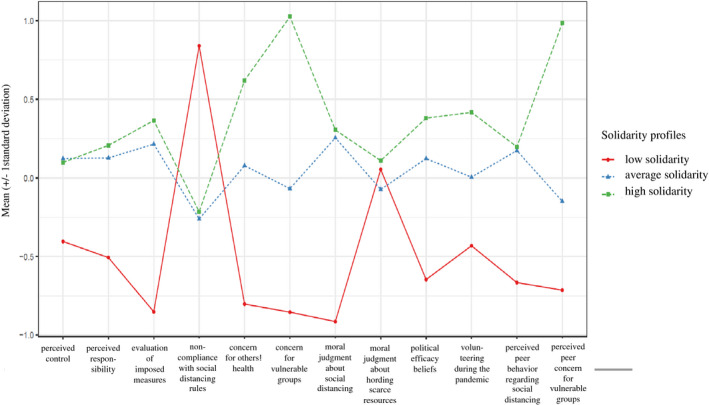
Latent solidarity profiles of young adults. *Note*: All variables were mean centered as they were on different rating scales. The *Y*‐axis represents the average level of the sample, plus and minus 1 *standard deviation*

When increasing the number of profiles, a three‐profile model fit the data better than a two‐profile model (except for the LMR test, see Table 3). Based on statistical indicators, a four‐profile model also fit the data well, while this was not the case for a model with five profiles (see Table 3). When comparing the fit indices of the three‐ and the four‐profile models, the BIC value was nearly the same. When examining the profile plots (showing the mean values of each variable within each profile), the three‐profile model consisted of three mostly quantitatively different profiles (i.e., low, average, and high levels of solidarity, see Figure 1). When inspecting the four‐profile model (for details, see Supporting Information, S3), the average solidarity profile was further differentiated into two different profiles; however, the differences between these two profiles were small. As our aim was to not only identify solidarity profiles but also identify predictors of solidarity in adolescence, we decided for the three‐profile model (given that it best explained the heterogeneity in the data and considering the relatively small sample).

**TABLE 3:** Fit information of the latent profile analysis and growth mixture models

No. of classes	Log likelihood	BIC	Entropy	LMR *p*‐value	BLRT *p*‐value
LPA					
1	−4836.67	9809.91			
2	−4582.53	9443.89	.80	.014	.000
3	−4501.22	9423.53	.82	.158	.000
4	−4427.91	9419.16	.80	.095	.000
5	−4380.52	9466.64	.83	.614	.020^a^
GMM sympathy					
1	−2076.62	4186.64			
2	−1940.36	3934.16	.70	.000	.000
3	−1927.31	3928.09	.80	.004	.000
GMM trust					
1	−2052.60	4138.61			
2	−1959.12	3971.68	.48	.004	.000
3	−1914.35	3902.18	.64	.016	.000
4	−1904.30	3902.11	.70	.041	.000
GMM peer exclusion					
1	−2507.50	5048.40			
2	−2389.83	4833.09	.81	.031	.000
3	−2348.39	4770.25	.76	.044	.000
4	−2328.92	4751.36	.79	.257	.000

Abbreviations: BIC, Bayesian information criterion; BLRT, bootstrap likelihood ratio test; GMM, growth mixture modeling; LMR, Lo–Mendell–Rubin Test; LPA, latent profile analysis.

^a^ Value may not be trustworthy and should be considered with caution.

The three‐profile solution (minimal profile probability = .89 and maximum profile probability = .93) consisted of three distinct and theoretically cohesive latent Covid‐19 solidarity profiles, mostly differing in the level of solidarity (see Figure 1): low (23%), average (54%), and high solidarity (23%). Young adults in the low solidarity profile perceived low control, low responsibility, and low efficacy regarding the measures to prevent the spread of Covid‐19, and did not comply with social distancing measures. This group also did not perceive it as wrong to disregard social distancing measures. Similarly, young adults in this group were low on all other community‐related measures, except for the judgment about hoarding essential resources, where their value aligned at mean level with the other two groups. Lastly, those in the low solidarity profile perceived that their friends did not comply with social distancing rules and expressed low levels of sympathy for people at risk.

In contrast, young adults in the average solidarity profile have average values in all variables, except that their non‐compliance with social distancing measures was slightly below average, while their evaluation of the wrongfulness of not complying with social distancing rules was slightly above average. The high solidarity group was not only quantitatively but also qualitatively different from the average profile, as young adults in this group reported much higher concern for others’ health, higher concern for people affected by Covid‐19, and higher perceived peer concern than the average group. Moreover, they had slightly higher values regarding political efficacy and volunteering.

### Predictors of latent profile membership: Variable‐centered approach

First, we analyzed solidarity profile membership with the three‐step approach by using the BCH weights in MPLUS (Asparouhov & Muthén, 2014), whereby we excluded non‐significant predictors in order to increase power. In these analyses, we also controlled for gender, migration background, and parental education; however, since no significant associations with the profiles were found, the control variables were dropped for this analysis in order to increase power. The results (see Table 4) showed that adolescents at age 18 with higher levels of sympathy were significantly less likely to be in the low or average solidarity profile compared to the high solidarity profile at age 20. Moreover, higher levels of sympathy at age 12 were also significantly associated with a lower likelihood to belong to the low, respectively, to the average profile relative to the high solidarity profile. For social trust, higher levels at age 18 predicted a lower likelihood to be in the low versus high solidarity profile (i.e., they were 3.75 less likely to be in the low relative to the high solidarity profile). Lastly, adolescents with higher reported peer exclusion at age 18 were more than twice as likely to belong to the average solidarity as compared to the high solidarity profile.

**TABLE 4:** Prediction of profile membership to the latent solidarity profiles with the predictors sympathy, trust, and peer exclusion

	Low solidarity *n* = 67	Average solidarity *n* = 161
	coef (log‐odds)	coef (log‐odds)
Sympathy T1	−1.09 (0.34)*	−0.92 (0.40)*
Sympathy T3	−1.88 (0.15)**	−1.10 (0.33)*
Trust T3	−1.27 (0.28)**	−0.17 (0.84)
Peer exclusion T3	0.63 (1.88)	0.83 (2.29)*
AIC	520.38	
BIC	557.25	

Note

The model displays the coefficients of the latent profile analysis with the three‐step BCH‐method (Asparouhov & Muthén, 2014) and the respective results from the MLM regression with the high solidarity profile as reference category (*N* = 67). The exponentiated coefficients of the MLM models in brackets express the likelihood of belonging to the other solidarity profiles relative to the high solidarity profile for an increase of 1 unit in the predictor variable. T1 = age 12 and T3 = age 18. In order to increase power, non‐significant predictors (including the control variables) were deleted in a stepwise procedure. The final model is shown.

Abbreviations: AIC, Akaike information criterion; BIC, Bayesian information criterion; MLM, multinomial logit model.

*p < .05, **p < .01, two‐tailed.

**FIGURE 2:**
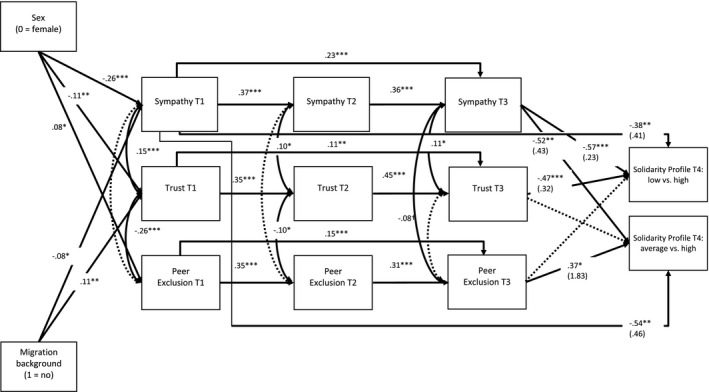
Structural equation model predicting classification into the different solidarity profiles. *Note*: Standardized coefficients are reported for all associations between variables. For the associations of the predictor variables with the latent profiles, the log‐odds are reported below the standardized model coefficients in brackets

The SEM (see Figure 2, *df* = 51, AIC = 12,673.97, and BIC = 12,911.72; fit indices without profile membership as outcome variable, but including control variables: *χ* ^2^ = 110.47 [*df* = 42], *p* = .000, comparative fit index = 0.93, root mean square error of approximation = 0.05 [0.04, 0.06], and standardized root mean square residual = 0.05), controlling for the stability of each predictor and for the within‐time associations (i.e., correlations between variables at each time point), showed similar results. While revealing relatively high stability from ages 12 to 18, the predictors at age 18 were significantly associated with the classification into the solidarity profiles. The model coefficients and associated log‐odds, exposing the likelihood of being classified into either the low or average as compared to the high solidarity profile, were very similar to the values obtained by the three‐step approach.

Taken together and in line with our hypotheses, both methodological approaches revealed that relative differences (i.e., on a continuous scale) in sympathy, social trust, and peer exclusion at earlier ages significantly predicted solidarity during the Covid‐19 pandemic. Moreover, sympathy in early adolescence was already significantly associated with solidarity at the age of 20. However, with regards to peer exclusion, our hypothesis was only partially supported as the contrast between the low and high solidarity profile was not significant.

### Predictors of latent profile membership: Exploratory person‐centered approach

#### Sympathy

For sympathy, a model with three latent growth trajectory classes fit the data better than a model with two classes (see Table 3). However, when considering class sizes, the third class in the three‐class solution only consisted of two cases. We, therefore, continued with the two‐class solution, differentiating between a class (79%) with relatively higher levels at age 12 (latent intercept = 3.89) and small decreases over time (latent slope = −0.04; high stable) and a class with moderate levels at age 12 remaining stable (intercept = 3.04, slope = −0.05, 21%; moderate stable). Overall, changes in sympathy from ages 12 to 18 were very small. When inspecting the cross‐table (see Table 5) of the sympathy growth trajectory classes with the solidarity profiles, the *χ* ^2^‐test suggested significant differences between the expected and the observed frequencies, *χ* ^2^(4) = 20.04, *p* < .001. If participants were more likely classified in the high‐stable sympathy trajectory, they were more frequently classified in the average and high solidarity profiles relative to when they were in the moderate‐stable sympathy profile.

**TABLE 5:** The overlap between latent growth trajectories of the predictors and the latent solidarity profiles of young adults

Frequency, row %, column %	Solidarity profile (*n* = 296)		
	Low (*n* = 68)	Average (*n* = 161)	High (*n* = 67)
Sympathy trajectory			
Moderate stable (*n* = 57)	25, 44%, 37%	27, 47%, 17%	5, 9%, 7%
High stable (*n* = 239)	43, 18%, 63%	134, 56%, 83%	62, 26%, 93%
Trust trajectory			
High stable (*n* = 110)	17, 15%, 25%	61, 56%, 38%	32, 29%, 48%
Moderate decrease (*n* = 175)	43, 25%, 63%	98, 56%, 61%	34, 19%, 51%
Low decrease (*n* = 11)	8, 73%, 12%	2, 18%, 1%	1, 9%, 1%
Peer exclusion trajectory			
High decrease (*n* = 9)	4, 44%, 6%	4, 44%, 2%	1, 12% 2%
Moderate decrease (*n* = 78)	16, 21%, 24%	45, 58%, 28%	17, 21%, 25%
Low stable (*n* = 209)	48, 23%, 70%	112, 54%, 70%	49, 23%, 73%

#### Social trust

When investigating trust, a solution with three growth trajectory classes showed a better fit than a two‐class model. However, a four‐class solution seemed also plausible (and had a similar BIC value to the three‐class solution, see Table 3). However, as the fourth class of the four‐solution model only included 10 participants, we continued with a three‐class solution. The first class (64%) had moderate levels of trust in early adolescence (intercept = 2.66) but decreased over time (slope = −0.21). The second class (5%) had relatively low values at age 12 (intercept = 2.00) and decreased more than the first class over time (slope = −0.40), while the third class, making up 31% of the sample, was characterized by high initial levels of trust (intercept = 3.11) and showed only slight decreases over time (slope = −0.04). When inspecting the cross‐table (see Table 5), the pattern suggested that participants in the high‐stable trajectory were relatively frequent in the high solidarity profile compared to the other growth trajectory classes, with participants in the moderate‐decreasing class being most frequent in the average solidarity profile and low‐trusting participants with a decreasing trajectory overrepresented in the low solidarity profile. The significant *χ* ^2^‐test suggested that the expected frequencies were different from the observed ones, *χ* ^2^(4) = 21.22, *p* < .001.

#### Peer exclusion

For peer exclusion, a solution with three growth trajectory classes fitted the data better than a two‐class one. This was not the case for a four‐class solution (see Table 3). The first class (4%) consisted of a minority of participants who experienced very high levels of peer exclusion (intercept = 3.68 and slope = −1.41), but strongly decreased over time. The second class consisted of 22% of the sample with moderate levels of peer exclusion, whereby exclusion slightly decreased over time (intercept = 1.75 and slope = −0.28). The third class (74%) showed low levels of peer exclusion in early adolescence and remained stable over time (intercept = 0.60 and slope = 0.02). When inspecting the cross‐table (Table 5), the few participants within the high peer exclusion trajectory were almost all categorized into the low and average solidarity profiles. Participants with moderate and low levels were most likely in the average and high solidarity profiles. The *χ* ^2^‐difference test was not significant though, *χ* ^2^(4) = 2.97, *p* = .563.

Taken together, the pattern of results suggests that the growth trajectories were associated with the solidarity profiles: trajectories with lower initial levels that remained stable or increased were more likely represented in the average and high relative to the low solidarity profiles. Given the relatively small sample size, these results are only descriptive.

### Validation of the solidarity profiles in an additional sample of adolescents

In order to validate the identified solidarity profiles in the sample of young adults, an additional sample of younger Swiss adolescents (*N* = 401, *M*_age_ = 16.28, range = 14–19, 31% male, 31% with a migration background) was examined. For this sample, a solution with three profiles fit the data best, whereby 19% of adolescents were in a profile characterized by low solidarity, 50% in a profile of average, and 31% in the profile of high solidarity. When inspecting the profiles, the patterns were very similar to those obtained for the young adults’ sample, although some smaller differences emerged. For political efficacy beliefs, evaluation of respective measures, and judgment about social distancing, differences were relatively larger between the high and average solidarity profiles as compared to the young adults’ sample. Concern for vulnerable groups and volunteering was also higher in the low solidarity profile of adolescents than in the low solidarity profile of young adults, whereby concern for vulnerable groups was still relatively lower than in the average and high solidarity profiles.

Taken together, while small differences emerged in the three profiles identified for both samples, the main pattern of results was replicated, adding to the validity of the profiles obtained. Detailed analyses can be found in the Supporting Information (see S4).

## DISCUSSION

### Young adults’ solidarity during the Covid‐19 pandemic

This study provides insights into how young adults respond to social challenges by highlighting different profiles of subgroups of young adults characterized by low, average, or high solidarity during the pandemic. Framing solidarity with the relational developmental systems’ metatheory (Lerner et al., 2014), we defined solidarity as a mutually beneficial individual–context relation and, therefore, as *adaptive* developmental regulation. Contrariwise, low solidarity or the absence thereof indicates *maladaptive* developmental regulation. The findings of this study show that about one quarter of the adolescents deviated from the average sample in their solidarity responses by either expressing high or low solidarity.

These novel findings on variation in young adults’ solidarity response help to understand how they adapt to challenging situations characterized by strong restrictions to their social life. While peers would be important for their identity development and social belonging (Andrews et al., 2020), young adults need to curb their social life, as they can transmit the virus to vulnerable groups while they are often asymptomatic (Davies et al., 2020). Thus, for the sake of longer‐term alleviation of severe health complications, overcrowded hospitals, and other social and economic consequences, the findings show that, except for about one quarter of the sample, young adults accepted shorter‐term restrictions to personal freedom.

Moreover, this study adds to the understanding of how pandemic‐related solidarity can be characterized. In line with our assumption that this construct would reflect a complex, multifaceted response, involving interrelated components, the findings of the latent profile analyses revealed three mostly quantitatively different profiles, varying in the level of disease‐related, community‐related, and peer norm‐related components. As the hypothesized components of the multifaceted solidarity construct were clearly interrelated for different groups of young adults, the current study highlights which specific components require further attention in future research when examining differences in solidarity. Furthermore, knowing in particular how rather high and rather low solidarity are expressed by young adults during the pandemic provides insights into specific components that could be addressed to foster solidarity, even if they are mostly quantitatively different.

For example, while solidarity profiles of young adults with average and high solidarity did not differ much in disease‐related components, they did so in the low solidarity profile (expanding the work by Nivette et al., 2021). Additional target areas for this group would be *community‐related components* of solidarity, whereby, for example, young adults with low solidarity do not perceive the response of the government as effective (in line with findings by Alessandri et al., 2020). By adding additional solidarity components to this rather scarce previous work on pandemic‐related solidarity, the current study, thus, provides multiple avenues for future research on this topic.

One integral component strongly differentiating the young adults in the low solidarity profiles from the other profiles pertained to their moral judgment of what correct behavior during a pandemic ought to be with regard to social distancing. This finding aligns with previous work on the significance of socio‐moral beliefs for adolescents’ contributions to the common good (Metzger et al., 2019). Relatedly, the low solidarity profile differed from the higher ones in the moral judgment about social distancing, but not regarding the hoarding of essential resources. Thus, the minimal standard of fair behavior during a health crisis seems to be the moral condemnation of unfair distribution of essential resources. The different judgments of these two components are in line with the assumption of various domains of social knowledge regarding different aspects of civic engagement and moral conflicts (Metzger et al., 2019; Turiel, 2006). Finally, the conflict of negative consequences for others’ health versus the strong need for personal affiliations in young adulthood may be more difficult to navigate with respect to social distancing measures compared to avoiding the hoarding of essential resources. Future research may, thus, assess young adults’ reasoning about different conflicting needs during a pandemic.

When inspecting differences between groups of young adults, perceived peer norms of pandemic‐related behaviors were an additional important component, which was interrelated with the other components of solidarity within the profiles. Thus, the current study also contributes to perceptions of peer behaviors as reference points for pandemic‐related solidarity. In particular, while perceiving peers as rule neglecting may negatively shape young adults’ beliefs about Covid‐19 and social distancing behavior, perceiving peers as expressing high concern for vulnerable groups may foster volunteering and expressing concern for vulnerable groups. Although the findings of the current study cannot be interpreted causally (as they were part of cross‐sectional profile analyses), they nevertheless align with previous work on positive (e.g., volunteering; Choukas‐Bradley et al., 2015) and negative peer influences (e.g., rule‐neglecting behavior; Dahl & van Zalk, 2014; Veenstra et al., 2018). However, several limitations apply: First, as this was an anonymous online study, peer norms were operationalized as individual perceptions. Such perceptions may not be as accurate in measuring peer behavior and depend on other aspects of the relationship, such as social preference (e.g., Prinstein & Wang, 2005). Thus, future work could include a measure of both perceived and actual peer norms and peer behavior. In addition, this study did not investigate mechanisms by which perceived peer behavior shaped individual attitudes and vice versa (peers may also select others with similar behavior and attitudes); future research may clarify these processes with regards to solidarity.

In addition to the mostly quantitative differences between profiles discussed, a few qualitative differences emerged. In particular, individual and perceived peer concern for vulnerable groups set the high solidarity profile qualitatively apart from the other ones, suggesting that for the high solidarity group, individual and perceived peer concern were strongly correlated. Hence, concern for others seems to be an integral part of solidarity as adaptive developmental regulation and aligns with previous conceptualizations of solidarity focusing on considerations for others, such as the need of holding together and standing in for each other (e.g., Bierhoff & Küpper, 1999). Furthermore, these concern‐related components can also inform discussions about improving solidarity in policy and practice.

### Adolescent predictors of young adults’ solidarity in times of crisis

This study also explored whether developmental processes from early to late adolescence (i.e., ages 12–18) related to sympathy, social trust, and peer exclusion would be conducive to young adults’ adaptive developmental regulation shown as solidarity in a pandemic (i.e., age of 20). The findings pointed to the importance of all three predictors, although there were different patterns of associations with later solidarity. We discuss the implications of these findings for devising strategies to promote solidarity in a pandemic.

#### Sympathy

Results revealed that sympathy in late adolescence was a robust predictor of young adults’ solidarity, even when taking the level of sympathy developed in early and mid‐adolescence (i.e., ages 12 and 15) into account. Remarkably, over and beyond the stability, the level of sympathy at age 12 significantly predicted solidarity at age 20. These findings add to the scarce longitudinal evidence that sympathy in early adolescence is a developmental precursor for investing in the common good in late adolescence as expressed in social justice attitudes and informal helping (Grütter & Buchmann, 2021). Corroborating these findings, participants with a high‐stable sympathy trajectory from early to late adolescence were more frequently classified in the average and high solidarity profiles relative to when they were in the moderate‐stable sympathy trajectory. However, it must be acknowledged that sympathy trajectories only differed with regards to their initial level; thus, sympathy seemed to be characterized by high stability in early adolescence, further adding to this important time window in early adolescence.

Taken together, these findings support the notion that the development of intentions and behaviors that benefit the common good rests upon normative developmental processes from early to late adolescence. To promote solidarity in young adults for coping with tomorrow's global crisis, the takeaway message for practice is that fostering the development of sympathy early on will pay off in later solidarity.

#### Social trust

Late adolescents with higher levels of social trust were nearly three times and significantly less likely to be in the low versus high solidarity profile as young adults, even when taking the considerable stability of social trust from early to late adolescence into account. While trust measured at earlier ages was not *directly* associated with later solidarity profiles, participants’ trust trajectories from early to late adolescence revealed that those with high and stable trajectories (31%) belonged more frequently to the high solidarity profile compared to the other two trajectory growth classes. Congruent with the normative age‐related decline in social trust (Flanagan & Stout, 2010), almost two thirds of participants (64%) with moderate levels of social trust in early adolescence did so. They belonged most frequently to the moderate solidarity profile later on, while the few ones (5%) with low and declining trust values were almost exclusively categorized into the low solidarity profile.

From a practice perspective, interested in promoting young adults’ solidarity in times of crisis, these findings suggest that promoting social trust in late adolescence can foster solidarity in young adults. Moreover, when also taking the descriptive findings from the trajectory analyses into account, fostering high levels of social trust in early adolescence and slowing its decline across adolescence may result in higher solidarity in young adulthood.

#### Peer exclusion

Results showed that adolescents with higher levels of peer exclusion at age 18 belonged significantly more likely to the average solidarity profile compared to the high one. Thus, peer exclusion experienced in late adolescence seemed more relevant for solidarity than that from early adolescence. However, adolescents with higher levels of peer exclusion were not significantly more likely to be in the low rather than in the high solidarity profile as young adults. This finding is contrary to previous work showing that experiences of peer exclusion increase the risk of developing deviant behavior and affiliating with more deviant peers (e.g., Dishion & Tipsord, 2011). From a statistical point of view, our sample size limited the power to detect an effect of smaller magnitude; therefore, the current result should be replicated with a larger sample size, particularly as the analyses suggest that effects of peer exclusion experienced at the age of 18 on profile membership may be rather small. Moreover, no significant association between the peer exclusion trajectories and solidarity profile membership was found. However, our sample size was too small to allow for solid conclusions regarding the group with high peer exclusion. It could be that particularly high levels of exclusion in early adolescence pose a risk factor for the expression of later solidarity. Previous work showed that high and chronic levels of exclusion are associated with lower levels of school liking and school safety as well as higher levels of school avoidance (Ladd et al., 2017; Nylund et al., 2007). Thus, these adolescents may be at higher risk for later deviant behavior. Thus, future research is warranted that sheds more light on the complex associations between peer experiences over a longer period of time and pay attention to moderating and mediating competencies and peer affiliations that may protect adolescents who suffer from peer exclusion from disengagement toward the common good later on.

### Limitations and future directions

The findings of this study reflect young adults’ solidarity expressed in the early phases of the pandemic. The question, therefore, arises, whether the identified solidarity profiles would remain robust with the pandemic's progression. This is important, as Switzerland, after lifting the first lockdown in late spring 2020, was among the few Western countries pursuing a path of relatively moderate pandemic‐related measures. This changed toward the end of 2020, when more strict measures were introduced, culminating in a second lockdown. Research should clarify whether the duration of the pandemic would provoke symptoms of fatigue, particularly among young adults expressing average solidarity during the first lockdown, potentially resulting in growing membership in the low solidarity profile.

Moreover, although this study used a sample permitting longitudinal predictions based on panel data, our sample size was small, restricting the power to differentiate our analyses even more (i.e., number of profiles; association between profiles to be analyzed with latent growth models). Thus, the findings should be replicated in future studies with different samples (Spurk et al., 2020). A first validation of the solidarity profiles was provided with the additional sample of Swiss adolescents, who filled in a similar questionnaire as the young adults. Nevertheless, since these findings point to potential developmental differences in the relative importance of the solidarity components, future research is needed. In addition, replications with samples collected in different countries would be interesting and add to the generalization of our conclusions as the virus affected adolescents in different contexts differently and different containment measures were imposed (Brauner et al., 2020).

While the current sample can speak for a country strongly affected by Covid‐19 during early phases of the pandemic (Salathé et al., 2020), it is nevertheless a context characterized by a well‐organized health and social system. Thus, additional work from samples with similarly high case numbers and fewer resources may reveal a more comprehensive picture of how young adults cope with the challenges of solidarity. Thereby, a particular focus on social minority groups would be of interest, as they may have been differentially affected by Covid‐19 and measures to contain the spread of the virus.

Another limitation of the current study was that the measures relied on self‐reports of young adults. Still, the relatively high stability and scalar measurement invariance of the predictor measures over a period of 8 years speak for the reliability and validity of the measures. For the variables included in the solidarity profiles, the nature of social restrictions made it difficult to assess data other than in an online format. Future research could, thus, include data from other sources (e.g., peers, parents, and teachers) and rely on electronic data (e.g., data on physical mobility) in order to further validate the findings of the current study.

## CONCLUSION

The current study provided new insights on whether and how young adults express solidarity during the Covid‐19 pandemic. By conceptualizing solidarity as multidimensional construct, three groups of young adults characterized by either low, average, and high solidarity were identified. While facing strong restrictions to their social life, only a minority of young adults expressed low solidarity. In order to promote adaptive development within this group, comprehensive strategies may need to be developed, such as, addressing their sense of control and responsibility, their trust in governmental responses, fostering their concern for vulnerable groups, and positive peer perceptions.

In addition, it is also important to consider how future generations can be prepared to contribute to the common good in future health crises. Thereby, as the current work suggests, sympathy already developed in early adolescence may benefit solidarity in adulthood. This integral social competence helps to promote actions for the welfare of others and provides an early target point for promoting young adults’ adaptive development. In addition, as social trust and peer exclusion, particularly during more recent phases of development, are relevant for expressing solidarity, adolescents’ social connection and belonging represent additional goals for promoting adaptive development, potentially fostering social connectedness and solidarity in future crises.

## CONFLICT OF INTEREST

The authors declare that they have no conflict of interest.

## Supporting information

Supplementary Material
