# Supplementary material for: Developmental antecedents of young adults’ solidarity during the Covid‐19 pandemic: The role of sympathy, social trust, and peer exclusion from early to late adolescence
Source: Child Dev. 2021 Aug 31;92(5):e832–50. doi: 10.1111/cdev.13660 (PMC8653140; doi:10.1111/cdev.13660)
Supplement: Supplementary file 1 — Supplementary Material [file CDEV-92--s001.doc]

Supplemental Materials for:

Developmental Antecedents of Young Adults’ Solidarity during the Covid-19 Pandemic: The Role of Sympathy, Social Trust, and Peer Exclusion from Early to Late Adolescence

**S1 Full list of measures**

A full list of all measures is displayed in Table S1.

**S2 Measurement Invariance**

Since our modeling strategies included investigating mean-level changes of constructs over time, we tested three nested models of longitudinal measurement invariance (Millsap, 2011): (1) configural invariance in which the same set of fixed and free factor loadings (*λ*) was specified across time (T1, T2, and T3) without any equality constraints; (2) metric invariance in which factor loadings of the same item were constrained to equality over time (e.g., *λ* item1 at T1 = *λ* item1 at T2 = *λ* item1 at T3); and (3) scalar variance, in which the intercepts of the same items are constrained to be equal over time (e.g., τ item 1 at T1 = τ item 1 at T2 = τ item 1 at T3). When metric invariance holds, regression coefficients and covariances can be properly interpreted. Scalar variance in turn is needed to be able to interpret mean-level changes.

To test whether the equality constraints imposed were tenable, we considered a nonsignificant delta chi-square test (Δχ²) or a ΔCFI with a threshold of .01 (ΔCFI ≤ 0.01; Cheung & Rensvold, 2002) as indicative of longitudinal metric invariance. To scale our latent variables, we fixed their variances at 1 and we freely estimated their factor loadings. All analyses were run using maximum likelihood estimation of the parameters (MLR) in M*plus* 8 (Muthén & Muthén, 2018). As reported in Tables S2, partial scalar invariance was reached for all predictor variables. This means that at least one intercept was not constrained to equality over time (Cheung & Rensvold, 2002). We used modification indices to identify sources of differences, whereby for the respective indicator that was not invariant over time, the specific constraint was relaxed (Little, 2013). All indicators showed positive and statistically significant factor loadings on their intended latent factor.

**S3 Solution with Four Solidarity Profiles**

When inspecting the solution with four solidarity profiles (see Figure S3), the mid-category was subdivided into two categories, while the low and high solidarity profile patterns remained the same. In the two average profiles, one group showed the highest moral judgment regarding compliance with social distancing (compared to all other profiles) and higher restriction of social contacts, as well as higher perceived effectiveness of the measures to contain the spread of COVID-19 and higher levels of solidarity than the lower average profile (and low solidarity profile). In contrast, the lower average profile was above the mean value with regard to meeting friends (i.e., complying less to social distancing measures) and slightly below the mean with regard to all other aspects (except for the moral judgment regarding hoarding essential resources). In this solution 14% (*n* = 43) belonged to the low solidarity profile, 32% (*n* = 96) to the lower average solidarity profile, 32% to the higher average solidarity profile (*n* = 97), and 20% to the high-solidarity profile (*n* = 60).

**S4 Validation of the Solidarity Profiles in an Additional Sample of Adolescents**

In order to validate the identified solidarity profiles during the Covid-19 pandemic, an additional sample of adolescents from the Swiss German-speaking part (*N* = 401, M_age_ = 16.28, range = 14-19, 31% male, 31% with a migration background, whereby 74% were from European countries, 17% from Asia, 4% from Latin America, 3% from Africa and 2% from Northern America) was analyzed. These adolescents answered an online questionnaire with the same questions as the sample of young adults of the COCON study. They received the link for the voluntary participation on the platform where the materials for the online schooling were provided. The assessment was cross-sectional.

The results of the latent profile analyses (conducted in MPLUS) showed that a solution with 2 and 3 profiles fit the data well, while according to the LMR-test, a solution with 4 profiles did not fit to the data significantly better than the solutions with fewer profiles (see Table S4). The 3-profile solution had a lower BIC and lower Log-Likelihood value than the 2-profile solution and showed high entropy. When considering the distribution of adolescents into the profiles, 19% were in a profile characterized by low solidarity, 50% in a profile of average and 31% in the profile of high solidarity.

When inspecting Figure S4, showing the means of the different groups on the dimensions of solidarity, the pattern of results is very similar to the profiles obtained in the sample of young adults, with similar relative differences between the groups. Some smaller deviations were that adolescents in the high solidarity profile evaluated the imposed measures as more effective and not complying with social distancing measures as more wrong than adolescents in the average solidarity group (in contrast to the profile of young adults where they both had similar values).

When looking at average differences between the young adults’ and adolescents’ profiles obtained from the two samples, adolescents in the average and high solidarity profiles were characterized by higher political efficacy beliefs than young adults. Moreover, when considering the low solidarity profile of the adolescent sample, their concern for vulnerable groups and volunteering was higher than in the low solidarity profile of young adults, whereby concern for vulnerable groups was still relatively lower than in the average and high solidarity profiles.

Taken together, while small differences emerged in the three profiles that were identified in both samples, the main pattern of results was replicated, adding to the validity of the profiles obtained. Still, future research could more closely expect potential age differences between adolescents’ and young adults’ solidarity, whereby the relative importance of some aspects may vary depending on age.

Table S1

***Descriptive Statistics of the Predictor and Outcome Variables / Scales of the Solidarity Profiles***

| **Predictors** | **Components of the Covid-19 Profiles** | | | | |
| --- | --- | --- | --- | --- | --- |
|  | |  |  | | |
| **Sympathy**  “When I see someone being picked on, I feel sorry for them.”  “I feel sorry for children who can't afford to buy so many things.”  “I feel sorry for other children who are sad or in trouble.”  “When I see another child doing badly, I feel sorry for him or her.” | | **Perceived Control***  “Not much can be done against Corona.”  **Perceived Responsibility**  “Whether I get Corona or not, depends on how I behave.”  **Evaluation of Imposed measures**  How helpful are the following strategies in preventing the spread of Corona? (0 very ineffective, 5 = highly effective)  “Meet fewer friends”, “Avoid crowds of people”, “Wash hands regularly and disinfect”, “Keep a distance of 2 meters”, “Stay at home” | |  | |
| **Trust**  “Most people take advantage of others when  they have the opportunity.”*,  “Most people can be trusted.”  “Most people try to be fair.”  “Most people think of their own advantage.”*  **Peer Exclusion**  “I sometimes get picked on by peers” “I sometimes get excluded by peers.” | | **Non-Compliance with Social Distancing Rules**  “How many friends have you personally met last week in total?”  (number of friends the person met during the lockdown)  **Concern for Others’ Health**  I am worried…  ”...that my friends or my family could become seriously ill because of Corona.”  “...that I could infect my friends or my family with Corona.”  “...that, around the globe, many people will fall sick.”  “...by the fact that people will become seriously ill if I don’t follow the rules.”  “…that Corona continues to spread.”  “…Corona will come back again.” | |  | |
|  | | **Concern for Vulnerable Groups**  I am concerned …  “... about elderly and sick people who can hardly go out.”  “... about elderly and sick people who are alone.”  “...about elderly and sick people who severely suffer from the consequences of Corona.”  “… about people who have to work much more than usual because of Corona.”  “... about people in other countries who have too little to eat because of Corona.” |  | | |
|  | | **Moral Judgment about violating Social Distancing Measures* (high values reflect not accepting the violation)** | | |  |
|  | | In times of the Corona-virus, is it ok or not ok if I… (0 = completely not ok, 5 = completely ok)  “…continue to individually meet my friends?” “…take the bus or tram during my free time?” “…go to a party of my friends?” “…hang out with my friends outside as a group?” |  | | |
|  | | **Moral Judgment about Hoarding Scarce Resources * (high values reflect not accepting the violation)**  In times of the Corona-virus, is it ok or not ok if I… (0 = completely not ok, 5 = completely ok)  “…buy more medicine than I need?” “ “...buy more toilet paper than I need?” |  | | |
|  | | **Political Efficacy Beliefs during the Pandemic**  “The Federal Council has the situation under control.”  “The Federal Council is doing a good job regarding Corona.”  “The Federal Council has issued rules that are too strict.”*  “The Federal Council does not care what people like me think.”*  “I trust the Federal Council.”  “The Federal Council has too much power.”* |  | | |
|  | | **Volunteering during the COVID-19 Pandemic**  How often do you volunteer presently? (0 = I don’t, 4 = daily)  “I help my family in the household (e.g. shopping/gardening/cleaning/cooking etc.).”  “I help older people and other risk groups in the household (e.g. shopping/garden).”  “I help older people or risk groups by calling them to see how they are doing (e.g. call grandparents regularly).” |  | | |
|  | | **Perceived Peer Behavior Regarding Social Distancing During the Pandemic** |  | | |
|  | | “My friends follow the rules about social distancing released by the Swiss government.” |  | | |
|  | | **Perceived Peer Concern for Vulnerable Groups** |  | | |
|  | | “My friends are concerned about elderly and sick people who can hardly go out.” “My friends are concerned about elderly and sick people who are alone.” “My friends are concerned about elderly and sick people who severely suffer by the consequences of Corona.” |  | | |

*Note.* W = Wave. If no specific scale is written, the young adults answered the items on a 6-point scale ranging from 0 = completely disagree to 5 = completely agree. Items with * have been recoded to build the scale. Scales with * have been recoded to build the profiles.

Table S2

*Measurement Invariance across Time (i.e., age 12 to 18) of the Predictor Variables*

|  | χ^2^ (df) | CFI | MC | Δ χ^2^ (Δ df), *p* | | Δ CFI |
| --- | --- | --- | --- | --- | --- | --- |
| **Sympathy** |  |  |  |  | |  |
| 1. Configural | 53.060 (39) | .996 |  |  | |  |
| 2. Metric | 75.840 (45) | .991 | 2 vs. 1 | 22.780 (6), *p* <.001 | | .005 |
| 3. Scalar | 393.352 (51) | .901 | 3 vs. 2 | 317.512 (6), *p* < .001 | | .090 |
| 4. Partial scalar | 86.811 (48) | .989 | 4 vs. 2 | 10.971 (3), *p* < .05 | | .002 |
| **Social Trust** |  |  |  | |  |  |
| 1. Configural | 132.387 (36) | .953 |  | |  |  |
| 2. Metric | 144.256 (42) | .950 | 2 vs. 1 | | 11.869 (6), *p* > .05 | .003 |
| 3. Scalar | 208.148 (48) | .922 | 3 vs. 2 | | 63.892 (6), *p* < .001 | .028 |
| 4. Partial scalar (time and cohort) | 157.618 (45) | .945 | 4 vs. 2 | | 13.357 (3), *p* < .05 | .005 |
| **Peer Exclusion** |  |  |  | |  |  |
| 1. Configural | 0.992 (3) | 1.00 |  | |  |  |
| 2. Metric | 0.992 (3) | 1.00 | 2 vs. 1 | | ­ | 0 |
| 3. Scalar | 69.420 (5) | 0.95 | 3 vs. 2 | | 68.428 (2), *p* < .001 | 0.05 |
| 4. Partial scalar | 0.992 (3) | 1.00 | 4 vs. 2 | | – | 0 |

*Note.* df = degrees of freedom. CFI = Comparative fit index. MC = model comparison. Δ χ^2^ = χ^2^ difference test. *Δ* CFI = Change in CFI. *Δ* CFI and was computed by subtracting the CFI value of the more constrained model from the null-model (see MC).

Figure S3. *Latent Solidarity Profiles – Four Profile Solution*

*Note.* All variables were mean-centered as they were on different rating scales. The y-axis represents the average level of the sample, plus and minus 1 standard deviation.

Table S4

***Fit Information of the Latent Profile Analysis with the Adolescent Sample***

| *No of classes* | *Log Likelihood* | *BIC* | *Entropy* | *LMR p-value* |
| --- | --- | --- | --- | --- |
| LCA |  |  |  |  |
| 1 | -6488.73 | 13121.31 |  |  |
| 2 | -6076.15 | 12446.01 | .82 | 0.000 |
| 3 | -5948.92 | 12341.39 | .80 | 0.031 |
| 4 | -5878.83 | 12351.07 | .79 | 0.552 |

Figure S4. *Latent Solidarity Profiles – Adolescent Sample*

*Note.* All variables were mean-centered, as they were on different rating scales. The y-axis represents the average level of the sample, plus and minus 1, respective 1.5 standard deviations.

**References**

Cheung, G. W., & Rensvold, R. B. (2002). Evaluating goodness-of-fit indexes for testing measurement invariance. *Structural Equation Modeling: A Multidisciplinary Journal*, *9*, 233–255. doi:10.1207/S15328007SEM0902_5

Little, T. D. (2013). Longitudinal structural equation modeling. New York, NY, US: Guilford Press.

Muthén, L. K., & Muthén, B. O. (2018). *Mplus User’s Guide.* (7th ed.). Los Angeles, CA: Muthén & Muthén.
